# Diet, Society, and Economy in Late Medieval Spain: Stable Isotope Evidence From Muslims and Christians From Gandía, Valencia

**DOI:** 10.1002/ajpa.22647

**Published:** 2014-10-29

**Authors:** Michelle M Alexander, Christopher M Gerrard, Alejandra Gutiérrez, Andrew R Millard

**Affiliations:** 1 BioArCh, Department of Archaeology, University of YorkYork, UK; 2 Department of Archaeology, Durham UniversityDurham, UK

**Keywords:** C_4_ plants, Islamic, Mediterranean, collagen, faith

## Abstract

This article investigates the diets of neighboring Christians and Muslims in late medieval Spain (here 13th–16th centuries) through the analysis of the stable isotopes of carbon (δ^13^C) and nitrogen (δ^15^N) in adult human and animal bone collagen. Twenty-four Christians and 20 Muslims are sampled from two adjacent and contemporaneous settlements in the township of Gandía on the Mediterranean coast, together with the remains of 24 animals. Statistical differences in both δ^13^C and δ^15^N reveal that the diets of the two faith communities differed, despite living side-by-side. These differences may relate to inequalities in their access to foodstuffs, particularly to C_3_/C_4_ grain and/or possibly terrestrial meat sources, though cultural preferences are also highlighted. Isotopic values for animals were also found to vary widely, both between and within species, and this provides a window into the local livestock economy. Am J Phys Anthropol 156:263–273, 2015. © 2014 The Authors. American Journal of physical Anthropology published by Wiley Periodicals,Inc.

Stable isotope analysis of archaeological bone collagen is a well-established technique that can provide direct evidence of the diet of archaeological populations down to the level of an individual (Ambrose, 1993). This information needs to be properly contextualized to maximize its benefits and is best used alongside other sources of evidence such as faunal assemblages and written texts. A growing number of projects have now applied the technique to medieval Mediterranean populations (e.g., Salamon et al., 2008; Bourbou et al., 2011; Fuller et al., 2012; Reitsema and Vercellotti, 2012; Ciaffi et al., 2013), but major published studies from Spain remain restricted to the Balearic Islands (Fuller et al., 2010; Nehlich et al., 2012). Responding to this challenge, this article presents data from late medieval populations from the mainland peninsula and examines the diets of Muslims and Christians living in a single locale in the Kingdom of Valencia during the 13th–16th centuries AD. It is the first such study to attempt this.

The intermingling of faiths in medieval Spain can be explored from numerous angles, through literature, architecture, the development of science and the study of archaeological objects, to name a few (e.g., Mann et al., 1992). The study of diet using stable isotope evidence is particularly noteworthy, however, because it offers the prospect of detecting directly the dietary preferences of different faiths living side-by-side within the same community. In brief, Christians, Muslims and Jews co-existed in the Iberian Peninsula for much of the Middle Ages, at first under Islamic political control in al-Andalus after the 8th century AD, and later under Christian rule after the ‘reconquest’ gained momentum during the 12th and 13th centuries. This life together, or *convivencia* as it is termed, lent itself to some surprising cultural influences, for example the popularity of couscous among Christians and their adopted habit of dining when seated on a cushion on the floor (de Castro, 1993: 172). However, it ended in the forced conversion and subsequent expulsion of Jews and Muslims from Spain after AD 1492 and 1609, respectively.

The realities of religious co-existence within this pluralistic society have long been the subject of academic debate (Glick, 1992; Soifer, 2009) and food, in particular, might be expected to gain a significance that went beyond physiological need. While dietary preference may be constrained by the local availability of foodstuffs, it is often used to express cultural and social identities such as status, ethnicity, gender, occupation and regional difference (e.g., Montanari, 1999; Super, 2002; Parker Pearson, 2003; van der Veen, 2003). Religious groups, in particular, may identify themselves by what they choose to consume or to avoid (MacClancy, 1992:42; Latham and Gardella, 2005). With this in mind, the aim of the research described here was to use stable isotopes to characterize the diet of two faith communities living in the same locality. To achieve this, human samples were obtained from Gandía (Valencia), a large urban settlement with good archaeological evidence for contemporaneous Christian and Muslim burials. Animal remains were also obtained to act as an isotopic baseline and in order to investigate aspects of economy and animal husbandry.

## Historical Background

**Figure 1:**
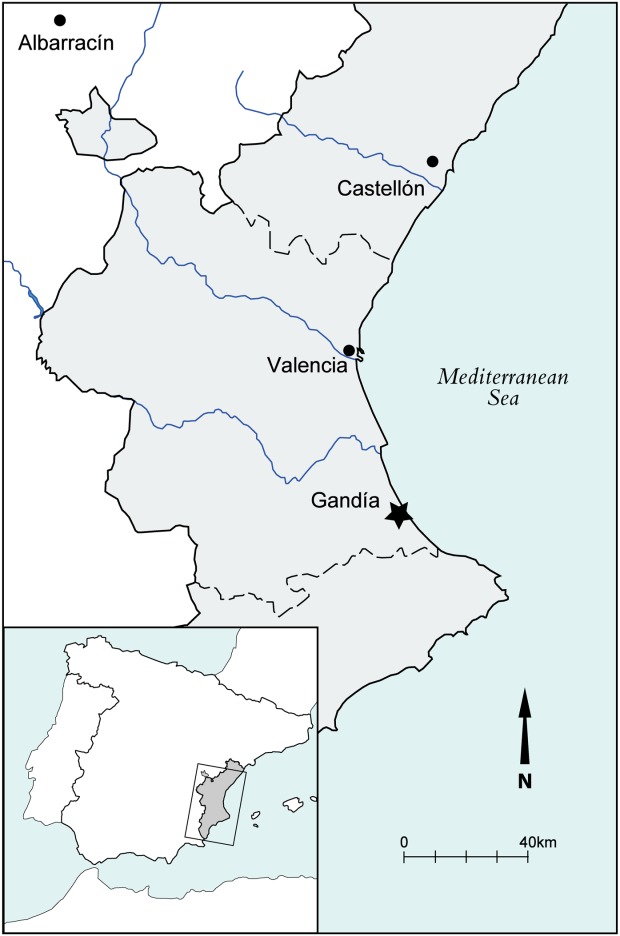
Location map showing the modern autonomous community of Valencia (in grey) and the location of Gandía and Albarracín (Teruel). [Color figure can be viewed in the online issue, which is available at http://wileyonlinelibrary.com.]

Gandía lies on the Mediterranean coast of Spain, around 65-km south of the modern city of Valencia (Fig. 1). Urban settlement first developed here in the 13th century after the Christian reconquest in 1249 and it is in this region of eastern Spain that the largest *mudéjar* population could be found during the later Middle Ages (Sesma, 2008); *mudéjares* being the remaining Muslim population living in territory controlled by the Christians, an ethnic minority characterized by its adherence to Islam. By the end of the 15th century these two faith communities had lived side-by-side for over 250 years, the Muslims being tolerated by the Christians, by now segregated and closed away from the religious perspective but, at the same time, making vital contributions to agriculture, traditional crafts and commerce (Guiral, 1989:435–504; Hinojosa, 2002). Gandía, by this date a royal duchy, was a successful commercial town with an economy rooted principally in the growing of sugarcane and the refining of sugar which was then exported to an international market (Guiral, 1989:424; Aparisi and Alonso, 2011). Archaeological excavation and historical research has shown that the town was surrounded by a cluster of small settlements in which the local population were *mudéjares*; one of these was at Benipeixcar, originally an *aljama*, or self-governing community of Muslims, which has since been absorbed into the modern town of Gandía.

In the early 16th century, the *mudéjares* were forced to convert to Christianity, and these “crypto-Muslims” were known as *moriscos*; many of them retained their Islamic faith in secret. This generated friction with the Christian community (Vincent and Ortiz, 1978; Ehlers, 2006) and the *moriscos* were finally obliged to depart in the early 17th century. In the meantime, they continued to perform an important role in the local economy, especially in the sugar industry as well as in the making of sweet confectionery (Meyerson, 1990:118, 134; Vincent, 1992:1). For brevity, the Muslim and later population of Benipeixcar of the 15th–16th centuries are referred to as *mudéjares* in this article, while the existence of various Spanish kingdoms has been simplified here by reference to “Spain.”

## Diet in Medieval Spain

The study of diet in medieval Spain is based on a wide range of available historical evidence. Taken together, these imply a diet based on cereals, with regional differences in the types of grain being consumed, as well as variation according to social status. Bread was made from unrefined wheat while grains such as barley and rye were eaten by those at the lower end of the social hierarchy together with C_4_ grains such as foxtail and broomcorn millet and sorghum in both red and white varieties (de Castro, 1993:286; García Marsilla, 1993:77, 259; García Sánchez, 2002:279; Tomás, 2009:466). Islamic texts also mention a long list of other grains or pulses for making bread of different qualities such as rice, barley, chickpeas and lentils (García Sánchez, 1995). Some of these grains are also referred to in historical texts as animal fodder (Rubio, 1995; Cortonesi, 1999) and archaeobotanical evidence confirms the presence of C_4_ millets at medieval sites further north in Catalonia, although always in relatively minor quantities when compared against other cereals (Alonso, 2005; Alòs et al., 2006−2007; Cubero et al., 2008). Although these grains will have formed a part of the diet of some proportion of the medieval population, current evidence falls short when considering their actual contribution. Another C_4_ grain, maize, can be ruled out as a potential source of C_4_ protein in this study. Maize was introduced to Europe after 1492 but historical evidence suggests it was not commonly grown in the Valencia region until the 17th and 18th centuries (Gómez, 1974; Sauer, 1976: 824; Crosby, 2003:179).

Other foodstuffs were supplied from the kitchen garden like onions, garlic and legumes such as beans, chickpeas, lentils and peas. To these should be added a great variety of fruit such as pomegranates and oranges, honey, eggs, and dairy products such as milk and cheese which supplied additional protein. Meat was widely consumed but the type, quality, and quantity depended on status, geography, and faith. Oxen, for example, were considered to be the food of the poor (García Sánchez, 1983:139; de Castro, 1993; Tomás, 2009:466) whereas meat from young and suckling animals was a high status food in demand on the urban market (Díaz, 1983; Martínez, 1996; Cortonesi, 1999). The most common types of meat consumed were mutton and lamb followed by kid, chicken, pork (in the case of Christians), beef, and game such as rabbits (Waines, 1992; de Castro, 1993; García Sánchez, 1996). Fresh fish would have been an important supplement to the diet along the coast and rivers as well as providing a catch for sale (de Castro, 1993). As many as 27 types of fish are recorded in local port books, from eels to sardines and tuna, and including also octopus (Guiral, 1989:388) while salted, dried, preserved fish (*escabeche*) and smoked sea-fish were also widely consumed and traded far inland (Gallart et al., 2005); sardines, for example, were imported to the Valencia region from Galicia in northern Spain (Ferreira, 1988:732). Remains of cuttlefish (*Sepia officinalis*) and possibly crab or crayfish from nearby sites in Alicante confirm that cephalopods and crustaceans also probably formed part of the Islamic diet (Benito, 1987).

Fish fulfilled a liturgical requirement for Christians; fast days (typically Wednesdays and Fridays) and Lent together accounting for over 150 days in the Christian calendar (Tomás, 2009:465), although other foodstuffs could be substituted for meat, among them cheese, eggs, legumes, nuts or vegetables (García Marsilla, 1993:76; Adamson, 2004:189; Rodrigo, 2009:571; Grumett and Muers 2010:26–27). There are nuances to this generic description however, the Church in Spain, for example, allowed the consumption of eggs and dairy products in Lent officially only after 1491 (Tomás, 2009:465) while monasteries and religious orders had their own fasting practices, some prohibiting meat entirely (Sesma, 1977:67; Grumett and Muers 2010:43).

Muslims were prohibited from consuming pork and any animal that had not been slaughtered in such a way as to be considered halal but, in terms of fasting restrictions, Islamic dietary law was actually less restrictive than the dietary regimes prescribed by Judaism and Christianity in the strictest sense (Insoll, 1999:95–97; Zaouali, 2007). In that context it is interesting that some remains of pig were recovered from Benipeixcar. Zooarchaeological evidence indicates that the presence of pigs is not unusual for Muslim sites of this period and the very few remains that are recovered are usually attributed to the presence of a small number of Christians on site or else to the hunting of wild boar (Morales et al., 1988; Lentacker and Ervynck, 1999; Antunes, 1996; Benito, 2006; Davis, 2006). This reminds us that, although some correlation between foodstuffs and faith is expected, much of the medieval diet will be influenced by what was affordable and available locally, thereby demonstrating cultural affinities and social status, as well as religious practices.

In summary, while there are still relatively few detailed published studies of well-dated faunal assemblages from later medieval sites in Spain, we nevertheless have a reasonable impression of the foodstuffs available. We can say far less, however, about what was consumed by whom and in what proportions and this complicates any fuller examination of the differences in diet between faith communities. Stable isotope analysis, on the other hand, offers exactly that opportunity because it can examine the diet of individuals, and the technique is also well suited to applications in regions where C_4_ plants were cultivated, as they were in Gandía.

## Reconstructing Diet Using Stable Isotope Analysis

Carbon (δ^13^C) and nitrogen (δ^15^N) isotopes in bone collagen are most frequently used for dietary reconstruction in archaeological populations; several reviews of the application of stable isotopes in archaeology are available (e.g., Katzenberg, 2000; Sealy, 2001; Schwarcz and Schoeninger, 2011). The isotopic composition of body tissues such as bone collagen reflects that of mainly the protein portion of the diet averaged over many years prior to death, although for low protein diets fats and carbohydrates may contribute carbon to the collagen (Hedges, 2004; Hedges et al., 2007) and different protein sources of animal origin such as meat and milk cannot be distinguished (O'Connell and Hedges, 1999). Of significance here is the fact that carbon isotopes can discriminate between plants that use different photosynthetic pathways; C_3_ plants include common crops such as wheat, barley and oats, most fruits, legumes and vegetables grown in temperate climates, whereas C_4_ plants are better adapted to hotter, drier environments, including tropical grasses and crops such as sorghum, millet, maize and sugarcane (O'Leary, 1981). C_4_ plants exhibit more enriched carbon values than C_3_ plants, with mean values δ^13^C of −13‰ and −27‰ respectively (Smith and Epstein, 1971; O'Leary, 1981). One confounding factor is the presence of marine foods more enriched in ^13^C than C_3_ terrestrial based resources (Schoeninger and DeNiro, 1984). As a result, carbon can be used to distinguish marine protein consumption in terrestrial C_3_-based diets but, when C_4_ plants are involved, marine and terrestrial carbon values can overlap (Sealy, 1997). Stable nitrogen isotope ratios, however, do help to distinguish between the consumption of purely marine protein or C_4_ plants, because the δ^15^N values of tissues provide information about the trophic level at which an organism is feeding; nitrogen is enriched by around 3–5‰ with each step along the food chain (Bocherens and Drucker, 2003; Hedges and Reynard, 2007). Carbon, on the other hand, exhibits less enrichment with values of 0–2‰ as trophic levels increase (Bocherens and Drucker, 2003). As aquatic foodchains tend to be longer than terrestrial ones, marine and freshwater resources usually exhibit higher δ^15^N values than terrestrial sources due to greater enrichment in nitrogen (Schoeninger et al., 1983). As noted elsewhere, however, this may not be the case in the Mediterranean as the ranges of δ^15^N values of marine and terrestrial animals from this region can overlap considerably (Craig et al., 2013). Terrestrial plants can also demonstrate elevated δ^15^N values as a result of natural environmental conditions such as salinity and aridity or anthropogenic factors such as the spreading of manure as fertilizer (Bogaard et al., 2007; Fraser et al., 2011).

There is some uncertainty in the reconstruction of the protein part of the diet of an individual using collagen δ^13^C and δ^15^N. Feeding studies indicate that there may have been underestimation in the magnitude of the diet-tissue offset in nitrogen with each trophic step (Δ^15^N_diet-collagen_), which may be up to +6‰ (O'Connell et al., 2012). Furthermore, due to macronutrient routing, there may be an unequal contribution of carbon from different dietary sources, which could lead to inaccuracies when estimating the amount of particular foods such as marine protein in archaeological populations (Craig et al., 2013). While precise reconstruction of an individual's diet is more problematic, carbon and nitrogen stable isotope analyses of bone collagen remains a powerful and informative tool for investigating relative differences between the protein parts of the diets of past populations and individuals.

## Samples and Methodology

Samples of adult human bone were taken from the burials of 20 *mudéjares* in an Islamic cemetery dating to the 15th to 16th centuries associated with the settlement of Benipeixcar (Fig. 1), excavated by J. Cardona in 1993–1994 (unpublished). All were buried SW-NE with the head facing east towards Mecca, in accordance with Islamic tradition. For comparison, 24 Christian skeletons were also sampled from three grave pits uncovered during excavations at the Colegiata de Santa María in nearby Gandía prior to restoration of the building (Vidal, 2006). The archaeological contexts from which these skeletal remains came are only broadly dated to between the 13th and 16th centuries, so there is a slight discrepancy in the date ranges of the *mudéjar* and Christian samples which cannot be resolved. Most importantly for this study, they represent two faith groupings from the same geographical location. Together with this human bone, terrestrial animal bones (*n* = 24) from a variety of species were also sampled from domestic contexts excavated within the settlement of Benipeixcar. The poor recovery of fish bones on site prompted the inclusion of additional marine fish from an 11th–12th century site at Albarracín in Teruel province (Fig. 1) in order to provide a contemporaneous isotopic baseline.

### Collagen extraction and stable isotope analysis

Rib bones were sampled from each adult human skeleton and cortical bone was chosen from animal bones, ensuring that only one sample was taken from each individual. Collagen was isolated from bone samples following a modified Longin (1971) method including an additional ultrafiltration step (Brown et al., 1988; Richards and Hedges, 1999). Samples of bone (∼0.2 g) were cleaned by air-abrasion and demineralized (0.5M HCl at 4°C for up to 10 days), the resulting insoluble fraction was gelatinized (HCl at pH 3) for 48 h at 75°C and the supernatant ultrafiltered to isolate the high molecular weight >30 kDa fraction which was then lyophilized. The purified collagen was analyzed in duplicate using continuous-flow isotope ratio mass spectrometry (CFIRMS) using a Delta XP mass spectrometer at either the Alaska Stable Isotope facility (University of Alaska Fairbanks's Water and Environmental Research Centre; WERC) for humans or at the Department of Human Evolution, Max Planck Institute for Evolutionary Anthropology (MPI-EVA, Leipzig, Germany) for animals. Isotopic values are reported here as the ratio of the heavier isotope to the lighter isotope (^13^C/^12^C or ^15^N/^14^N) as δ values in parts per mille (‰) relative to internationally defined standards for carbon (VPDB: Vienna Pee Dee Belemnite) and nitrogen (AIR) following the equation [δ*=* (*R*_sample_ *– R*_standard_)/*R*_standard_ × 1000] (Coplen, 1994). The instrument precision for δ^13^C and δ^15^N was ±0.2‰ (1σ) or better at both laboratories, determined by replicate analysis of internal laboratory standards. Five samples were compared between the laboratories yielding mean differences of 0.6‰ ± 0.2‰ (1σ) for δ^13^C and 0.0‰ ± 0.6 ‰ (1σ) for δ^15^N. The small difference in δ^13^C is insignificant at the magnitude of dietary interpretation discussed here. Sufficient collagen (>1%) was found to be preserved in all the samples and the collagen quality met published criteria (DeNiro, 1985; van Klinken, 1999). Statistical tests were performed using SPSS for Windows version 15. The non-parametric Mann–Whitney U and Kolmogrov–Smirnov tests for non-normally distributed data were employed.

## Results

**Figure 2:**
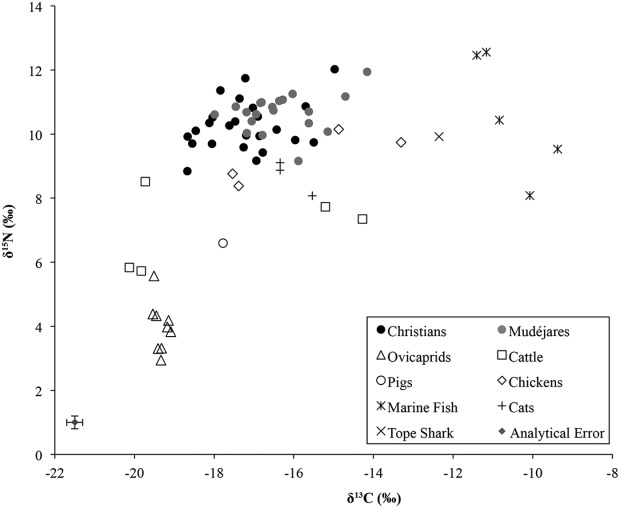
Plot of δ^13^C and δ^15^N values for Christians from the Colegiata, Gandía (13th–16th centuries); *mudéjares* and animals from Benipeixcar (15th–16th centuries); and marine fish from Albarracín, Teruel.

**Figure 3:**
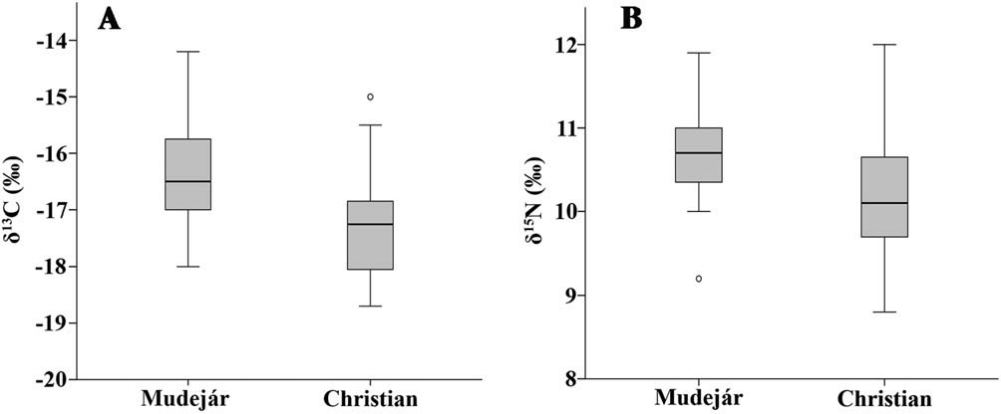
Boxplot comparison of δ^13^C (A) and δ^15^N (B) of *mudéjares* and Christians. Error bars denote 95% confidence interval, circles indicate outliers.

The human and animal stable isotope data are provided in Tables 1 and 2, respectively and plotted together in Figure 2; summary statistics for both humans and animals are listed in Table 3. An immediately noticeable feature of these results is that many of the humans in both populations register enriched carbon values suggestive of a measurable dietary input of C_4_ plants and/or marine protein. The most depleted δ^13^C values, on the other hand, suggestive of a largely C_3_ terrestrial-based diet, are exhibited by the Christians sampled from Gandía. Overall, despite the relatively small difference (<1‰) in absolute means for δ^15^N and δ^13^C between the populations of Christians (*n* = 24) and *mudéjares* (*n* = 20) shown in Table 3, the distribution of their δ^15^N and δ^13^C values is statistically different (two-sample Kolmogorov–Smirnov *z* = 1.486, *P* = 0.024, and *z* = 1.486, *P* = 0.024, respectively). Comparison in Figure 3 indicates that *mudéjares* demonstrate more enriched δ^13^C and δ^15^N values than their Christian contemporaries and this implies that the long-term diets of each group were significantly different.

**Table 1:** Results of stable isotope analysis of human bone collagen

Site	Sample	Sex	δ^13^C_VPDB_ (‰)	δ^15^N_AIR_ (‰)	C/N	%Col.
Colegiata; Christian (13th–16th centuries)	481	M	−15.7	10.9	3.3	2.8
	303	M	−15.5	9.7	3.3	4.4
	305	M	−15.0	12.0	3.3	3.4
	324	F	−16.0	9.8	3.3	3.5
	346	M	−18.1	10.4	3.4	5.9
	357	M	−16.8	9.4	3.3	7.7
	369	M	−17.2	10.0	3.3	4.4
	377	F	−18.7	9.9	3.4	2.4
	398	M	−17.5	10.4	3.4	5.2
	401	F	−17.6	10.3	3.3	2.7
	445	F	−18.6	9.7	3.3	7.7
	455	M	−16.4	10.1	3.4	1.4
	457	M	−18.7	8.8	3.4	2.2
	463	F	−17.3	9.6	3.3	5.2
	483	M	−17.4	11.1	3.4	2.9
	501	F	−16.9	9.9	3.3	3.8
	504	F	−16.9	9.2	3.3	3.2
	506	M	−18.0	10.5	3.4	1.8
	531	F	−18.1	9.7	3.3	5.5
	543	F	−17.0	10.8	3.4	8.6
	547	M	−18.5	10.1	3.4	2.3
	549	F	−16.9	10.5	3.3	2.7
	550	F	−17.2	11.7	3.3	2.9
	571	F	−17.8	11.4	3.3	4.1

Benipeixcar; *Mudéjar* (15th–16th centuries)	804	F	−17.2	10.0	3.3	7.3
	1504	M	−16.8	11.0	3.3	4.5
	1603	F	−14.7	11.2	3.2	9.5
	1904	?F	−15.9	9.2	3.3	2.6
	2003	F	−18.0	10.6	3.4	4.9
	2304	F	−16.3	11.1	3.3	5.1
	2404	F	−16.4	11.0	3.2	10.0
	2602	F	−16.5	10.8	3.3	9.4
	2703	?	−16.8	11.0	3.3	3.3
	2803	?F	−15.6	10.7	3.3	7.3
	2902	M	−17.2	10.7	3.3	4.6
	9203	M	−16.8	10.0	3.2	2.7
	9303	F	−16.5	10.7	3.3	5.2
	9503	M	−17.1	10.4	3.3	4.3
	9902	F	−16.0	11.3	3.5	2.5
	14403	M	−17.5	10.9	3.2	8.7
	14603	F	−16.9	10.6	3.4	2.5
	14703	F	−15.2	10.1	3.3	4.8
	15103	F	−14.2	11.9	3.2	7.9
	15403	F	−15.6	10.3	3.3	1.8

**Table 2:** Results of stable isotope analysis of animal bone collagen

Sample	Species	δ^13^C_VPDB_ (‰)	δ^15^N_AIR_ (‰)	C/N	% Col.
GBC1	*Bos taurus*	−15.2	7.7	3.3	1.5
GBC2	*Bos taurus*	−20.1	5.8	3.1	8.2
GBC3	*Bos taurus*	−19.7	8.5	3.2	4.1
GBC4	*Bos taurus*	−14.3	7.4	3.2	6.5
GBC5	*Bos taurus*	−19.8	5.7	3.1	11.1
GBCa1	*Felis Catus*	−15.5	8.1	3.2	6.2
GBCa2	*Felis Catus*	−16.3	9.1	3.2	2.7
GBCa3	*Felis Catus*	−16.3	8.9	3.2	2.8
GBF1	*Galeorhinus galeus*	−12.4	9.9	3.3	4.6
GBGa1	*Gallus*	−14.9	10.2	3.3	2.0
GBGa2	*Gallus*	−17.4	8.4	3.3	2.8
GBGa3	*Gallus*	−17.5	8.8	3.3	3.2
GBGa5	*Gallus*	−13.3	9.7	3.3	4.1
GBO1	*Ovicaprid*	−19.3	2.9	3.3	1.1
GBO2	*Ovicaprid*	−19.2	4.0	3.3	1.6
GBO3	*Ovicaprid*	−19.5	4.3	3.2	6.5
GBO4	*Ovicaprid*	−19.4	3.3	3.3	3.4
GBO5	*Ovicaprid*	−19.5	4.4	3.3	5.1
GBO9	*Ovicaprid*	−19.3	3.3	3.3	3.0
GBO6	*Ovicaprid*	−19.1	3.8	3.2	1.2
GBO7	*Ovicaprid*	−19.1	4.2	3.4	6.7
GBO8	*Ovicaprid*	−19.5	5.6	3.3	7.8
GBP1	*Sus*	−17.8	6.6	3.3	4.5
ABF1*	*Mugilidae Sp*.	−10.1	8.1	3.2	3.9
ABF5*	*Mugilidae Sp*.	−9.4	9.5	3.2	3.8
ABF6*	*Dicentratchus Sp*.	−11.4	12.5	3.2	6.2
ABF4*	*Dicentratchus Sp*.	−11.2	12.6	3.1	4.1
ABF3*	*Argyrosomus regius*	−10.8	10.4	3.0	16.2

Marine fish from Albarracín are marked with an asterix (*).

**Table 3:** Summary isotopic data for humans from the Colegiata de Santa María (Christian, 13th–16th centuries) and humans and animals from Benipeixcar (mudéjar, 15th–16th centuries)

Site/species	*n*	δ^13^C_VPDB_ (‰)				δ^15^N_AIR_ (‰)			
		Min	Max	Range	Mean ± 1σ	Min	Max	Range	Mean ± 1σ
Colegiata de Sta María	24	−18.7	−15.0	3.8	−17.2 ± 1.0	8.8	12.0	3.2	10.3 ± 0.8
Benipeixcar	20	−18.0	−14.2	3.7	−16.4 ± 1.0	9.2	11.9	2.8	10.7 ± 0.6
*Ovicaprids*	9	−19.5	−19.1	0.4	−19.3 ± 0.2	2.9	5.6	2.7	4.0 ± 0.8
*Bos taurus* (cattle)	5	−20.1	−14.3	5.8	−17.8 ± 2.8	5.7	8.5	2.8	7.0 ± 1.2
*Gallus* (chicken)	4	−17.5	−13.3	4.2	−15.8 ± 2.1	8.4	10.2	1.8	9.3 ± 1.8
*Sus* (pig)	1				−17.8				6.6
*Felis* (cat)	3	−16.3	−15.5	0.8	−16.1 ± 0.5	8.1	9.1	1.0	8.7 ± 0.5
*Galeorhinus* (tope shark)	1				−12.4				9.9

Standard deviations are shown when sample size is at least 3.

Comparison of human values with herbivores recovered from Benipeixcar reveals that human values from both sites are on average 2‰ higher in δ^13^C and 5.4‰ higher in δ^15^N than the means for herbivores (Table 3). The dietary spacing between humans and herbivores is wider than might be expected for trophic level enrichment (Bocherens and Drucker, 2003). This implies that both populations also consumed other enriched sources of ^15^N, perhaps chickens and their eggs, pork and marine fish, although those individuals with enrichment in both δ^13^C and δ^15^N values may have consumed marine fish in greater quantities. However, taking the potential dietary spacing for Δ^15^N_diet-collagen_ being 6‰ (O'Connell et al., 2012), the consumption of herbivore protein cannot be ruled out as a major dietary source.

More precise determination of individual dietary preference is complicated by several factors. First, isotopic values are distinct between species such as ovicaprids and cattle, implying different management strategies. However, there is also evidence of multiple management and feeding regimes for the same species. Two cows, GBC1 and GBC4, possess enriched carbon values of −15.2‰ and −14.3‰ indicative of a significant dietary intake of C_4_ plants when compared to other cattle which ate predominantly C_3_ diets. Two chickens, GBGa1 and GBGa5, also appear to have had a significant C_4_ plant contribution to their diet, with δ^13^C values of −14.9‰ and −13.3‰, respectively; C_4_ crops such as millet and sorghum, which make excellent feed for poultry, are likely to be responsible. Overall, however, the wide range of isotope values exhibited by the species in the sample makes it very difficult to determine the proportional contribution of different foods to the human diet, that is, marine foodstuffs, C_4_-fed herbivores, and C_3_-fed herbivores. This is particularly noteworthy in this case study because terrestrial fauna exhibit δ^15^N values that are similar to those of Mediterranean fish, which in turn tend to exhibit lower values than those elsewhere, in the Atlantic for example (Jennings et al., 1997; Garcia-Guixé et al., 2010; Barrett et al., 2011; Vika and Theodoropoulou, 2012; Craig et al., 2013).

## Discussion

### Economic and social explanations for dietary difference

Despite the inevitable uncertainties in determining diet, and the broad range of dates available for the Christian samples selected for this study, the differences observed between Christians and *mudéjares* in both δ^15^N and δ^13^C reflect their contrasting access to foodstuffs over long periods of their lives. In particular, the enriched δ^13^C values exhibited by *mudéjares* would imply that this Muslim or “crypto-Muslim” community relied more heavily on C_4_ plants, in their own diet and/or that of their animals.

This result is open to several possible interpretations. We have already noted that the *mudéjar* population of Gandía was closely involved with sugarcane cultivation and processing and it is probable that workers on the sugarcane plantations chewed on the raw canes to extract the sweet juice; indeed, inquisitorial records specifically indicate this was common practice among *mudéjares* in the 16th century (Galloway, 1989:16; Ehlers, 2006:96). This habit, however, cannot be the explanation for the elevated C_4_ signatures; sugarcane has an extremely low protein content and it would remain under-represented in the bone collagen provided that the diet contained adequate quantities of high protein foodstuffs such as meat. It is far more likely then that the adults with enriched carbon values obtained their C_4_ plant signatures indirectly through the consumption of animals foddered on bagasse, the fibrous waste from sugarcane crushing, and the green tops.

C_4_ crops, it should be noted, were generally regarded as a low-status foodstuff by society at this time; sorghum and millet were consumed only when the supply of other grains was lacking, particularly in rural settlements (Braudel, 1972:595; Glick, 1982:82; Sarasa, 1995:195; Adamson, 2002:8), so the observed dietary differences may reflect socio-economic disparities between rural (Benipeixcar) and urban (Gandía) and/or between *mudéjares* and Christian populations. Historical sources indicate the Duke of Gandía instructed the Muslim workers on his land to farm sugarcane due to its profitability and that this policy led to such a serious shortage of staple crops, such as wheat, that grain had to be imported from Valencia (Aparisi et al., 2009). A conventional interpretation of the results might therefore be that the Christian inhabitants of Gandía had privileged access to these wheat imports while the rural *mudéjar* population fell back on the cultivation and consumption of C_4_ crops. A similar emphasis on C_4_ crops has been noted among *mudéjar* populations in the Sierra de Espadán in the north of the Kingdom of Valencia in the 14th–16th centuries (Butzer et al., 1986: 372). Written records and archaeobotanical evidence indicate that communities there relied on the cultivation of millets and sorghum, and this has been linked to the “social segregation” of Muslims and, in particular, to a deterioration in their social standing following the Christian Reconquest (Ruiz, 2001). We should also remember that dietary differences between crypto-Muslims and Christians may have become more acute during the 16th century when halal meat became unavailable, at least legally, due to the prohibition of Muslim butchery practices (Vincent and Ortiz, 1978; Ehlers, 2006). Alternatively, it is also possible that the isotopic variation in both δ^13^C and δ^15^N is explained by a stronger preference for marine fish in the *mudéjar* diet. There is some historical evidence to suggest that meat rose in price in the Kingdom of Valencia during the 16th century, when the king prohibited the slaughtering of animals several times after 1525 (García Cárcel, 1977:100) and this may have led to a preference for fish, chicken and eggs among those of lower socio-economic status.

A word of caution is required, however, before these socio-economic interpretations are uncritically accepted. According to Islamic sources, millet (a C_4_ crop) was much appreciated by Muslims (Marín, 1997:10); it is mentioned, together with sorghum, in Islamic agricultural treatises (Hernández Bermejo et al., 1998:22) and it was cultivated together with wheat and barley in regions of the peninsula that lay outside Christian political control, such as in 13th-century Granada (Arié, 1992:158). Millet is also specifically listed in Islamic treatises as a licit product in which to trade (Constable, 1994:161) and this suggests that differences in cultural traditions in cooking and eating may also have had their part to play in the observed isotopic differences.

**Figure 4:**
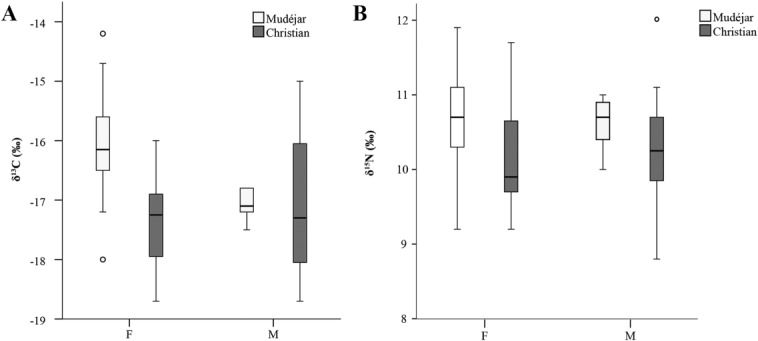
Boxplot comparison between δ^13^C (A) and δ^15^N (B) values and sex within the *mudéjar* and Christian populations. Error bars denote 95% confidence interval, circles indicate outliers.

The results presented here strongly suggest that the evident dietary differences between *mudéjares* and Christians originated in the home. A comparison of Muslim and Christian females reveals that the enrichment in ^13^C in particular is most pronounced in the *mudéjar* population (Mann–Whitney *U* = 21.5, *P* = 0.001; Fig. 4), although the observation of corresponding higher δ^15^N values among *mudéjar* females compared to Christian females is not statistically significant (Mann–Whitney *U* = 48.5, *P* = 0.067). If it can be assumed that the task of food preparation was, for the most part, taken up by women in medieval Christian and Muslim societies (Bynum, 1987:192; García Marsilla, 1993:152; Boone and Benco, 1999:67; Insoll, 1999:62–63), then this may have heavily influenced the difference in diet. Unfortunately, there were too few males (*n* = 5) in our *mudéjar* sample to explore this further and compare between males of different faith. The question might be resolved in the future by larger and better-balanced samples.

Turning finally to trends in diet *within* the two faith groups, statistical analysis indicates that there is no difference between the δ^13^C and δ^15^*N* values of males (*n* = 12) and females (*n* = 12) in the Christian population (Mann–Whitney *U* = 61.5, *P* = 0.544 for δ^13^C and *U* = 62.5, *P* = 0.583 for δ^15^N). This is not the same as saying that Christian households always ate together, only that their overall diet did not differ greatly or, at least, any differences are not observable isotopically with these sample sizes. Likewise, there is no written evidence to suggest that female and male *mudéjares* had a different diet; though special arrangements were made for women during pregnancy or lactation (Aubaile-Sallenave, 1997; García Sánchez, 2006). Again, the insufficient sample number prevents meaningful comparison between sexes among *mudéjares*.

### Implications for animal husbandry strategies at Gandía

This case study has also demonstrated significant differences in nitrogen values between ovicaprids and cattle. This may be due, on the one hand, to the stocking of cattle closer to settlements, where soils are likely enriched with manure and domestic refuse (Hedges et al., 2005; Bogaard et al., 2007; Commisso and Nelson, 2010). The sheep, on the other hand, may have been grazed further afield in transhumant flocks (Montalvo, 1992:162) as they were in the mountains of the Sierra de Espadán, north of Valencia, where there were both small, locally owned herds and large inter-regional flocks that grazed seasonally (Butzer et al., 1986).

The enriched carbon values exhibited by certain cattle probably reflect foddering on C_4_ plants such as millet, sorghum or sugarcane. In his 12th century *Treatise on Agriculture*, Ibn al'Awwam mentions that the by-products of sugarcane, among them molasses and discarded cane, supply excellent cattle fodder (Ibn al-Awwam, 1802) and this is exactly the practice in western India today where tops are fed green to dairy animals, as well as being dried and stored and fed like cereal straws (Mena, 1987; Yadar and Solomon, 2006). Given what we know of the local historic landscape, it seems highly likely that at least some of the C_4_ contribution to the diet of cattle came from sugarcane, specifically the draught animals put to work in the plantations and sugar mills. Other animals too had access to these C_4_ crops and while some animals, such as chickens and pigs, were foddered on millet, sorghum and/or sugarcane by-products; the cats possibly hunted rodents that would have scavenged C_4_ resources.

## Conclusion

Overall, the isotopic results presented here point to a complex, mixed C_3_/C_4_-based economy. The isotopic signatures of the animals sampled from Benipeixcar show a surprising degree of separation between species and within species, and our interpretation is that certain animals are likely to have grazed particular pastures and/or were deliberately fed significant quantities of C_4_ plants, a feeding regime which may be linked to their use as traction animals in sugarcane agriculture. Notably, the use of C_4_ crops in the study area was not restricted to cattle; chickens, cats and the single pig sample all exhibited enriched δ^13^C values that probably reflect the availability of C_4_ foodstuffs either through deliberate foddering or scavenging. While the use of C_4_ plants to fodder animals has previously been hypothesized for this period, as mentioned above, the data here represent some of the first clear isotopic evidence for this practice in medieval Europe.

We have also shown that both the Christian population from Gandía and the *mudéjares* from Benipeixcar consumed significant amounts of C_4_ plants directly and/or indirectly, with some individuals exhibiting an enrichment in both carbon and nitrogen which suggests an input of marine protein into their diets. In addition, there are significant differences in both carbon and nitrogen values between the two faith communities, indicating dietary differences which may be linked to the differing cultural and/or socio-economic status of the two populations. After weighing up the available historical and archaeological evidence, we have suggested that *mudéjares* on the whole may have eaten more fish from the Mediterranean and relied more heavily on C_4_ resources, stemming from their involvement in the cultivation and processing of sugarcane, a C_4_ crop, or from a cultural preference for particular grain types. Alternatively, their particular dietary signature may be the result of limited access to more desirable C_3_ plants, perhaps due to their declining socio-economic status at this time. Further clarification on this point could be sought through the application of single compound amino acid analysis (Corr et al., 2005; Choy et al., 2010) or in cases of excellent preservation, analysis of bone mineral apatite (Fernandes et al., 2012).

For the moment, while we cannot be certain about the food sources that underlie their dietary differences, we can demonstrate that the two contemporary faith communities ate different diets and begin to identify where those differences lay. Given the association of Christianity with fish consumption, it might be surprising that the Christians do not appear to have consumed more fish than the *mudéjares*, but perhaps this illustrates how differences in faith and culture can be subsumed by discrepancies in the regional availability of resources.

Our study illustrates well the challenge of teasing out medieval identities from archaeological data (Díaz-Andreu et al., 2005; Hinton, 2009). Dietary signals in isotopes intermesh food habits which may result from religious persuasion, gender, regional and community traditions, the availability of produce, kinship, economic grouping, and social ambition (Müldner, 2009); choosing between these and understanding how and why social groups selected some foodstuffs above others requires robust historical and theoretical underpinning, for example to understand the role of food in the social construction of memory (e.g., Jones, 1997; papers in Counihan and van Esterik, 1997; Sutton, 2001; Mintz and Du Bois, 2002). In that sense, this project is only a beginning. As yet we know little about how medieval diet differed between Spanish regions, the effects of social and economic change on eating patterns, the impact of racial laws and prohibitions, gender dynamics, food allocation, taboos, food symbolism, culinary memory, and much else besides. For 16th century Spain we should consider how many *mudéjares* there were in the region, the nature of the community and their contacts with Christians; not all populations were as embedded as the rural Valencian case study discussed here. There is a long chronology to reflect upon too; Valencia fell to the Reconquest in 1238 but Granada only in 1492, so *mudéjares* of the 16th century across the peninsula had very different experiences of Christian colonisation and conversion which would be interesting to compare. The “slow shipwreck” of Islam on the Iberian Peninsula (Braudel 1976, 781) is potentially one with an intriguing archaeological signature but there is still a considerable way to go before the full contribution of stable isotope techniques is fully realised.
